# Genotypic Analysis of the Population Structure in *Malassezia globosa* and *Malassezia restricta*

**DOI:** 10.3390/jof9020263

**Published:** 2023-02-15

**Authors:** Ines Hadrich, Nahed Khemakhem, Amin Ilahi, Houaida Trabelsi, Hayet Sellami, Fattouma Makni, Sourour Neji, Ali Ayadi

**Affiliations:** 1 Laboratory of Fungal and Parasitic Molecular Biology, School of Medicine, University of Sfax, Sfax 3029, Tunisia; 2 Faculty of Science, University of Gabes, Gabes 6029, Tunisia; 3 Laboratory of Parasitology—Mycology, UH Habib Bourguiba, Sfax 3029, Tunisia

**Keywords:** *M. globosa*, *M. restricta*, VNTR, clinical, animal, genotyping

## Abstract

The molecular characterization of *Malassezia* spp. isolates from animals and humans has not been thoroughly studied. Although a range of molecular methods has been developed for diagnosing *Malassezia* species, they have several drawbacks, such as inefficiency in differentiating all the species, high cost and questionable reproducibility. The present study aimed to develop VNTR markers for genotyping *Malassezia* isolated from clinical and animal samples. A total of 44 *M. globosa* and 24 *M. restricta* isolates were analyzed. Twelve VNTR markers were selected on seven different chromosomes (I, II, III, IV, V, VII and IX), six for each *Malassezia* species. The highest discriminatory power for a single locus was obtained with the STR-MG1 marker (0.829) and STR-MR2 marker (0.818) for *M. globosa* and *M. restricta*, respectively. After the analysis of multiple loci, 24 genotypes were noted among 44 isolates in *M. globosa*, with a discrimination index D of 0.943 and 15 genotypes were noted among 24 isolates in *M. restricta*, with a discrimination index D of 0.967. An endogenous infection was detected in two patients. Different genotypes of *M. globosa* strains colonized one patient. Interestingly, VNTR markers analysis revealed a carriage between a breeder and his dog in three cases for *M. globosa* and two for *M. restricta*. The FST (0.018 to 0.057) values indicate a low differentiation between the three populations of *M. globosa*. These results suggest a dominant clonal mode of reproduction in *M. globosa*. The typing of *M. restricta* showed a genotypic diversity of the strains, which can cause various skin pathologies. However, patient five was colonized with strains having the same genotype collected from different body parts (back, shoulder). VNTR analysis was capable of identifying species with high accuracy and reliability. More importantly, the method would facilitate monitoring *Malassezia* colonization in domestic animals and humans. It was shown that the patterns are stable and the method is discriminant, making it a powerful tool for epidemiological purposes.

## 1. Introduction

The genus *Malassezia* comprises lipophilic species that are normal parts of the human and animal skin flora. These yeasts are associated with various superficial dermatoses, including pityriasis versicolor, seborrheic dermatitis, folliculitis, and some variants of psoriasis and atopic dermatitis [1,2]. The genus *Malassezia* currently consists of 18 species [3], 10 of which have been isolated from human skin, including *Malassezia furfur*, *Malassezia pachydermatis*, *Malassezia sympodialis*, *Malassezia slooffiae*, *Malassezia globosa*, *Malassezia obtusa*, *Malassezia restricta*, *Malassezia dermatis*, *Malassezia japonica* and *Malassezia yamatoensis* [4]. *M. furfur*, *M. globosa* and *M. sympodialis* are the most commonly isolated species. *M. globosa* and *M. restricta* are the predominant species found on the scalp. *M. globosa* was found in pityriasis versicolor [5], while *M. restricta* was associated with seborrheic dermatitis and psoriasis [6,7,8]. The species distribution on the skin varies between different *Malassezia* related diseases, but their worldwide distribution may also differ. For example, *M. sympodialis* considered the most prevalent species in Europe and *M. restricta* and *M. globosa* were the most predominant species in Asia [8]. Since phenotypic identification methods are not very accurate for typing *Malassezia* species, various molecular techniques such as direct sequencing, PCR–restriction fragment length polymorphism (RFLP) [9,10], AFLP [11], RAPD [12] and real-time PCR [13,14] are gradually replacing the morphologic methods.

The Multiple Locus Variable-number tandem-repeat Analysis (MLVA) is based on the polymorphism of tandemly repeated genomic sequences called VNTR (Variable-Number Tandem-Repeats). VNTRs are classically separated into microsatellites (up to eight bp) and minisatellites (nine bp and more) [15]. The MLVA technique allows a resolution of closely related isolates for the investigation of disease outbreaks and provides information on the phylogenetic patterns among isolates [16,17,18]. The usefulness of MLVA was demonstrated for *Aspergillus fumigatus* [19], *Aspergillus flavus* [20,21], *Trichophyton interdigitale* [22] and *Candida parapsilosis* [23].

Here, we report the use of VNTR markers polymorphism as a new tool for the typing of *M. globosa* and *M. restricta* strains isolated from clinical and animal samples.

## 2. Materials and Methods

### 2.1. Fungal Isolates

A total of 68 *Malassezia* isolates were included in the present study: *M. globosa* (44) and *M. restricta* (24). *M. globosa* strains were collected from 26 patients (folliculitis (16), pityriasis capitis (6) pityriasis versicolor (4)) and 18 were isolated from breeders (9) and their dogs (9). Samples were collected using sterile compress from shoulder (7), face (8), neck (2), scalp (6) and back (10).

*M. restricta* strains were collected from 13 patients (folliculitis (5), pityriasis capitis (3) pityriasis versicolor (5)) and were isolated from breeders (7) and their dogs (4). Samples were collected using sterile compress from shoulder (3), face (7), neck (5), scalp (3) and back (2). Auricular samples from dogs were collected by swabbing.

Patients were referred to laboratory of parasitology mycology, UH Habib Bourguiba of Sfax-Tunisia.

All samples were then cultured on the modified Dixon agar medium and incubated for 3 to 7 days at 32 °C. These *Malassezia* strains were identified by real-time PCR, as described previously [14].

For the specificity tests, we used six *Malassezia* species [*M. globosa* (KU597270), *M. restricta* (KU597273), *M. pachydermatis* (KU597280), *M. slooffiae* (KU597272), *M. furfur* (KU597271) and *M. sympodialis* (KU597266)], four *Candida* species [*C. albicans* (ATCC 3153), *C. parapsilosis* (ATCC 2219), *C. glabrata* (CBS 138) and *C. tropicalis* (ATCC 66029)] and four filamentous fungi [*Aspergillus flavus* (MH329791)*, Aspergillus oryzae* (MH 675520), *Rhizopus arrhizus* (MH247234) and *Fusarium prolificans* (MH 329789)] for the specificity tests.

### 2.2. STR Design

We collected the published sequences of *M. globosa* and *M. restricta* genomes from NCBI databases (http://ncbi.nlm.nih.gov) (accessed on 26 January 2020). To identify the microsatellite and the minisatellite in *M. globosa* and *M. restricta* genome, we used the Tandem Repeats Finder software (http://tandem.bu.edu/trf/trf.html) (New York, NY, USA). For minisatellites, loci with tandem repeats consisting of more than 50 nucleotides and more than three repeats were selected. Six minisatellite and six microsatellite loci were finally selected. The Primer (version 3) software (http://frodo.wi.mit.edu) was used to design primers. BLASTn search (http://blast.ncbi.nlm.nih.gov/Blast.cgi) (accessed on 26 April 2020) was consulted to verify the specificity of *M. globosa* and *M. restricta* primers. Sequence manipulation suite (https://www.bioinformatics.org/sms2/) (Version 2, European Computer Manufacturers Association, University of Alberta, Edmonton, AB, Canada) was used to verify primers criteria selection.

### 2.3. DNA Extraction

DNA was extracted using a QIAamp DNA Mini Kit (QIAGEN, Hilden, Germany), as indicated by the manufacturer’s instructions. DNA concentrations were estimated with a spectrophotometer absorbance at 260 nm. DNA extracts were stored at −20 °C.

### 2.4. PCR Amplification of STR Markers

A multiplex-PCR amplification was performed in a final volume of 25 µL, containing 1 ng of genomic DNA, 5 µL of 5X reaction buffer (pH 8.5), 3 mM MgCl2, 0.2 mM (each) dATP, dCTP, dGTP and dTTP (Promega, Madison, WI, USA), 0.5 µM of each primer and 2.5 U of GoTaq DNA polymerase (Promega). The PCR was run on an Eppendorf thermocycler using these conditions: 94 °C for 5 min, 35 cycles of 94 °C for 30 s, 56 °C for 30 s, 72 °C for 1 min followed by 10 min at 72 °C. Microsatellite markers were amplified in PCR multiplex using 3 fluorescent labels (FAM, HEX and TET). PCR products were diluted tenfold with formamide. One microliter of the diluted amplification products was mixed with 15 µL of formamide and 0.5 µL of internal size marker LIZ 500 (Applied Biosystems Inc., Waltham, MA, USA). Following denaturation, the PCR products were resolved by capillary electrophoresis with polymer POP-7 in an ABI 3500 genetic analyzer (Applied Biosystems Inc., Waltham, MA, USA).

For minisatellite analysis, the amplification products of each minisatellite marker were then migrated on 3% agarose gel. The capture and analysis of the gels were performed by the Quantity One software package (Bio-Rad, Hercules, CA, USA) coupled to the imaging system Gel Doc XR^+TM^.

### 2.5. Data Analysis

Different DNA preparations of the same isolate with repeated analysis of the same DNA preparation were tested to evaluate the repeatability of the STR markers. We used the GENEPOP software version 4.2 to calculate genotype frequencies for each marker [24].

The Simpson index of diversity *D* was computed for each marker and each possible marker combination to determine the most parsimonious combination yielding a *D* value of 0.95, a sufficiently high discriminatory power recommended for typing experiments [25]. The genetic differentiation between both populations was measured using the pairwise fixation index (Fst) computed with the FSTAT V2.9.3 software [26]. To better visualize the relationship between the identified genotypes, we constructed a UPGMA dendrogram and a minimum-spanning tree performed using BioNumerics (version 7.6) software (Applied Maths, Sint-Martens-Latem, Belgium) [27].

## 3. Results

The use of the TRF program to study the distribution of VNTRs in the *M. globosa* genome allowed us to obtain 254 microsatellites and 467 minisatellites, of which 35.5% represent repeat motifs of one to eight nucleotides and the remainder (64.5%) represent repeat motifs greater than eight nucleotides. Using the TRF program to study the distribution of VNTRs in the *M. restricta* genome allowed us to obtain 120 microsatellites and 51 minisatellites, of which 85% represent repeat motifs of one to 12 nucleotides and the rest (15%) represent repeat units greater than 12 nucleotides.

Six microsatellites and six minisatellite markers were retained for further assays (Table 1).

No PCR product was generated after amplification of the 12 VNTR markers using the genomic DNA of four *Malassezia* species (*M. pachydermatis* (KU597280), *M. slooffiae* (KU597272), *M. furfur* (KU597271) and *M. sympodialis* (KU597266)), four *Candida* species (*C. albicans* (ATCC 3153), *C. parapsilosis* (ATCC 2219), *Candida glabrata* (CBS 138) and *Candida tropicalis* (ATCC 66029)) and four filamentous fungi *(Aspergillus flavus* (MH329791), *Aspergillus oryzae* (MH 675520), *Rhizopus arrhizus* (MH247234) and *Fusarium prolificans* (MH 329789)).

In addition, No PCR product was detected for *M. globosa* (KU597270) using *M. restricta* (KU597273) primers. The same is true for *M. restricta*. Thus, the 12 new loci were specific for the corresponding study: *M. globosa* and *M. restricta* (six for each).

For the specificity tests, we used six *Malassezia* species: *(M. globosa* (KU597270), *M. restricta* (KU597273), *M. pachydermatis* (KU597280), *M. slooffiae* (KU597272), *M. furfur* (KU597271) and *M. sympodialis* (KU597266).

Each marker’s reproducibility evaluated after repeated trials with different DNA preparations of the same isolate and with the same DNA preparation was 100%. These markers were specific to *M. globosa* and *M. restricta*.

### 3.1. Genetic Diversity

Upon the analysis of 44 *M. globosa* isolates, 3–6 distinct alleles were detected for each microsatellite marker. The highest discriminatory power for a single locus was obtained with the STR-MG1 marker, which had six distinct alleles and a *D* value of 0.829 (Table 1). The highest discriminatory power for a single minisatellite locus was obtained with the STR-MG4 marker, which had six distinct alleles and a *D* value of 0.807 (Table 1). The combination of three minisatellite markers yielded 15 different genotypes with a 0.886 *D* value.

The STR-MG6 marker exhibited the lowest diversity index (0.581) for *M. restricta* (Table 1). The highest discriminating power for a single microsatellite marker was obtained with the STR-MR2 marker, which had six distinct alleles and a *D* value of 0.818. The STR-MR6 marker exhibited the lowest diversity index (0.528). The STR-MR4 minisatellite marker (0.775) presented the highest discriminating power for *M. restricta* (Table 1).

After the analysis of multiple loci, 24 genotypes were noted among 44 isolates in *M. globosa*, with a discrimination index D of 0.943 and 15 genotypes were noted among 24 isolates in *M. restricta*, with a discrimination index D of 0.967.

### 3.2. Cluster Analysis

**Figure 1:**
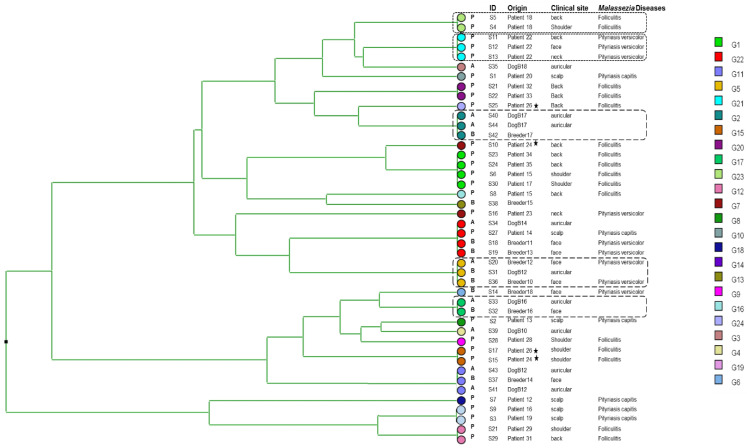
UPGMA dendrogram upon analysis of six VNTR markers obtained from 44 *Malassezia globosa* isolates. P: patient, A: animal and B: breeder. The highlighted isolates illustrate two epidemiological scenarios (S1 and S2): (S1) for P18 and P22 (square with dotted-line border), isolates collected from different clinical sites share the same genotype, indicating exposure to a clonal *Malassezia* population. For isolates highlighted by a square with a dashed-line border collected from breeders and their dogs present the same genotype indicating an animal–human co-transmission. (S2) P24 and P26 (star): each isolate has a distinct genotype, indicating exposure to a genetically highly heterogeneous clinical *Malassezia* population.

The dendrogram generated after the analysis of six markers for 44 isolates of *M. globosa* revealed genetic heterogeneity with 24 distinct genotypes. For instance, all four isolates sampled from patient 24 (P24) and patient 26 (P26) were genetically unrelated. However, we were able to identify a clonal cluster that included isolates collected from breeders and their dogs (S40, S44, S42/S20, S31, S36/S33, S32), indicating a possible co-transmission. We also noted that isolates from different patients share the same genotype (S23, S24, S6, S30/S17, S15/S9, S3/S21, S22) (Figure 1).

**Figure 2:**
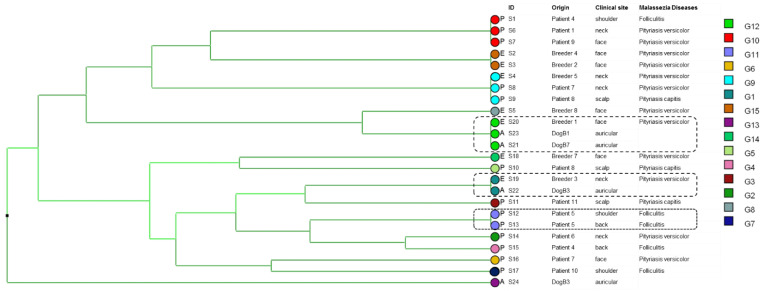
UPGMA dendrogram upon analysis of six VNTR markers obtained from 24 *Malassezia restricta* isolates. P: patient, A: animal and E: breeder. The highlighted isolates illustrate: for P5 (square with dotted-line border), isolates collected from the different clinical sites from the same patient share the same genotype. For isolates highlighted by a square with a dashed-line border collected from breeders and their dogs, present the same genotype indicating an animal–human co-transmission.

Typing study by the analysis of six new markers of *M. restricta* showed a great genotypic diversity of the strains, which can cause various skin pathologies. However, patient five (P5) was colonized with strains having the same genotype collected from different sampling sites (back, shoulder) and two breeders shared the same genotype of their dogs (Figure 2).

The calculated genetic diversity per locus (FST) values for the three populations of *M. globosa* strains ranged from 0.018 to 0.057, indicating weak differentiation between them (Table 2). These results suggest a dominant clonal mode of reproduction in *M. globosa*.

### 3.3. Interpopulation Genetic Analysis

**Figure 3:**
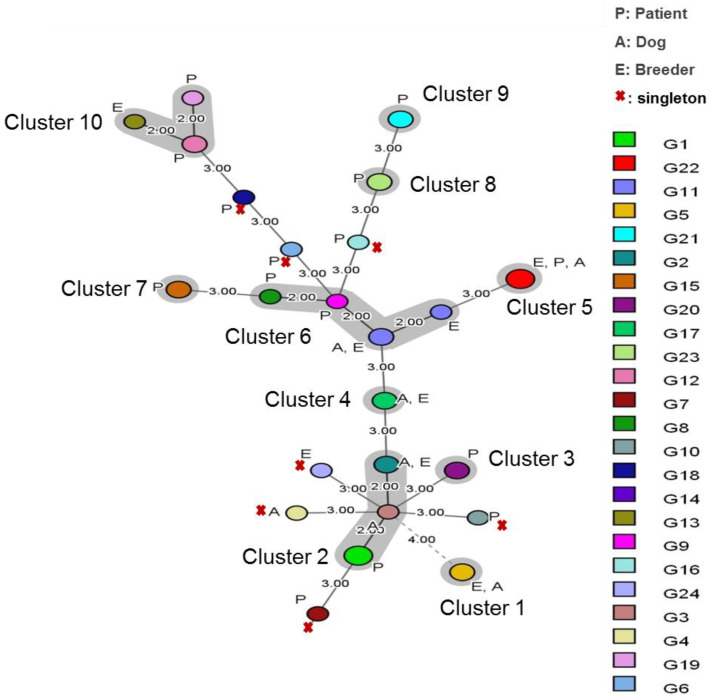
Minimum spanning tree showing the genotypic diversity of *Malassezia globosa* isolates (N = 44) based on VNTR data. Each circle shows a unique genotype and its size, the number of strains belonging to the same genotype. Connecting lines and numbers between circles show the similarity between genotypes: (1.00) indicates only one marker difference, (2.00) indicates a difference in two markers and (3.00) the difference in three markers.

An MST analysis of the 24 distinct multilocus microsatellite genotypes was performed to illustrate the genetic structure of our *M. globosa* population (Figure 3).

Genetic cluster analyses suggested that our *M. globosa* population was formed by ten genetic clusters; three different and distant clusters indicate differences in three markers. Cluster three, cluster seven, cluster eight and cluster nine contain exclusively strains from the patient’s isolates. Cluster one and cluster four contains isolates col-lected from breeders and dogs. Cluster two, cluster five and cluster six comprised *M. globosa* genotypes isolated in breeders, dogs, and patients. Cluster ten comprised *M. globosa* isolates collected from breeders and patients. Nine unique genotypes formed singletons (Figure 3).

**Figure 4:**
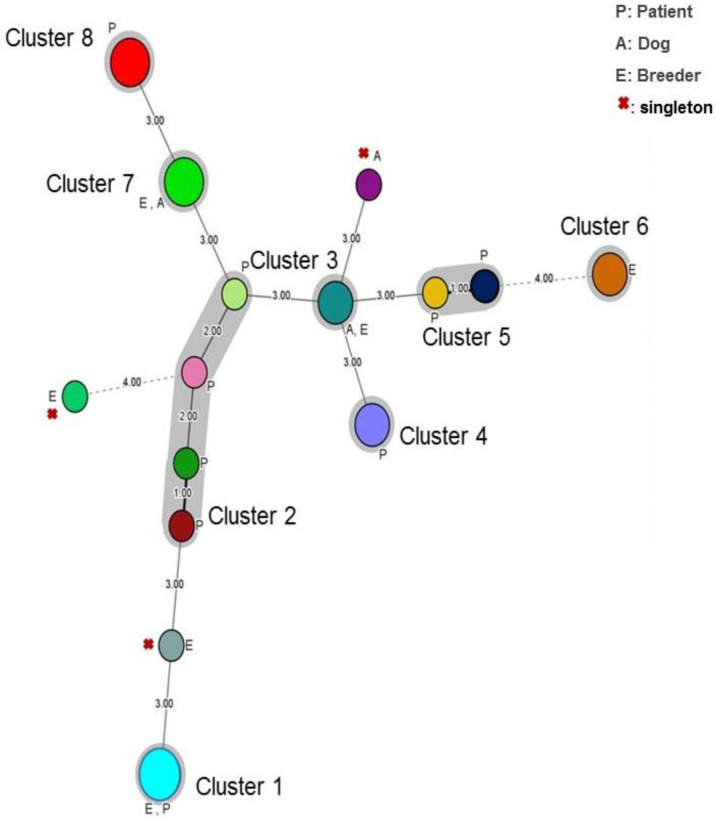
Minimum spanning tree showing the genotypic diversity of *Malassezia restricta* isolates (N = 24) based on VNTR data. Each circle shows a unique genotype and its size, the number of strains belonging to the same genotype. Connecting lines and numbers between circles show the similarity between genotypes: (1.00) indicates only one marker difference, (2.00) indicates a difference in two markers and (3.00) a difference in three markers.

The minimum spanning tree (MStree) generated for the 24 *M. restricta* isolates showed that eight distant clusters formed our *M. restricta* population. These clusters differed by three or four markers (Figure 4). The first cluster contains strains collected from the breeder and patients. The second cluster is dominated by strains collected from patients with different Malassezia diseases. Clusters four, five and eight contain *M. restricta* patient isolates. For cluster six, it is composed of *M. restricta* breeders iso-lates. Finally, cluster seven and cluster three contain strains isolated from the breeder and dog (Figure 4).

Three unique genotypes formed singletons. They were isolated from breeders or dogs (Figure 4).

The Chi-square test results of the covariates’ effect on the cluster distribution are presented in Table 3. The isolation site was not significantly associated with genotype clusters (*p* > 0.05). However, we noted a significative association of singleton genotype with strains isolated from patients with pityriasis capitis (*p* > 0.01).

Finally, the genotype clusters were significantly associated (*p* < 0.05) with the year when they were collected.

## 4. Discussion

Despite the recent increase in studies focusing on *Malassezia* sp., little is still known concerning *Malassezia* genetic diversity. Molecular typing of *Malassezia* strains and the elucidation of their genetic relationships are essential to address important epidemiological issues such as tracking the transmission of infection and strain specificity for a specific disease manifestation.

Few molecular methods have been used for typing *Malassezia* strains, such as RFLP and multigene sequence analysis [28,29,30]. In previous studies, various molecular techniques found genetic variability within *M. globosa*, *M. restricta*, *M. furfur* [31] and *M. pachydermatis* [32]. Different *Malassezia* genotypes were identified as strictly related to the host, geographical origin and/or clinical manifestations [31,33].

However, the major problem with such techniques is the poor interlaboratory reproducibility and difficulty in exchanging the results obtained by these techniques. To our knowledge, the level of discrimination between strains obtained with the microsatellite method has never been reached previously. Our study represents the first report on the development of stable and discriminatory microsatellite markers for typing *M. restricta* and *M. globosa*.

The microsatellite markers are characterized by a high level of polymorphism, discrimination, reproducibility and feasibility. Moreover, microsatellite analysis may be more able than other genotyping methods to distinguish between strains with low degrees of sequence variation.

The reproducibility and specificity of our markers were found to be 100%. Microsatellite analysis requires small amounts of DNA (~one ng per reaction) compared to other typing methods. Moreover, a large number of isolates may be typed by using multiplex assays with primers labeled with different fluorescent dyes, resulting in higher throughput.

Microsatellite polymorphism analysis revealed a heterogeneous *M. globosa* and *M. restricta* genetic populations. After multiple loci analysis, 24 genotypes were noted among 44 isolates in *M. globosa*, with a discrimination index D of 0.943 and 15 genotypes were noted among 24 isolates in *M. restricta*, with a discrimination index D of 0.967. Therefore, the new VNTR markers proposed in the present study have shown promising results in terms of the identification of polymorphism of *Malassezia* strains. Further analyses can clarify the role of this yeast in disease development and give us clues about the sexual reproduction that might provide genetic variation in this genus [34].

Hiruma et al. [35] found that specific genotypic strains of *M. globosa* and *M. restricta* predominated in patients with dandruff.

Czyzenwska et al. identified 17 distinct genotypes of *M. pachydermatis* with 59 polymorphic sites by sequencing analysis; however, putatively virulent strains were more closely related, indicating a probable correlation between the genotype and the virulence potential [32].

In our study, we found, by cluster analysis of *M. globosa* species, a similarity between strains collected from the different clinical sites of the same patient (patient 22, patient 18). This revealed that it is an endogenous infection, also called autoinfection; the patient becomes infected with his strains.

However, clonal infection was also observed in different patients (P17, P28, P34 and P35) sharing the same genotype.

Instead, we found a diversity of *Malassezia* genotypes from isolates collected from the different clinical sites. Multiple genotypes of a species can colonize the same patient [33,36,37], but some genetic types might be linked to a particular body site or pathology, thus indicating an affiliation of *Malassezia* genotypes with host, geographical origin and/or clinical manifestations [38,39].

For S21 and S23, two dogs are colonized by *M. restricta* strains which share the same genotype. This could be explained by horizontal transmission of strains with direct contact between individuals but also by exposure to common susceptibility factors associated with fungal colonization of the skin.

Clinical isolate and animal isolate have the same genotype, suggesting that *Malassezia* infection was acquired from the dog to the breeder. This highlights an exogenous infection which is a cross-infection transmitted between the breeder and the dog which can occur through direct contact. The animal-to-human or human-to-animal carriage is still not well known and its risk factors must be determined. Microsatellite analyses have been efficient epidemiological instruments to make clear the origins of *Malassezia* infections.

Intra-species variations in DNA pattern of *Malassezia* isolates and the presence of specific genetic types in cattle, dogs or humans were observed in others studies. Genetic heterogeneity of these yeast in veterinary and human medicine studies is given considering a possible transmission animal to human or human to animal [12]. Phylogenetic analysis would facilitate monitoring of *Malassezia* spp. carriage in domestic animals and in humans.

Cluster analysis of the 24 *M. restricta* isolates also showed a genotypic diversity, particularly in strains that can cause various skin pathologies. Soares et al. [40] founded 27 *Malassezia* subtypes using RFLP and sequencing, mostly from *M. restricta* (13 subtypes), indicating variation among species and also intra-specific polymorphisms. Subtypes of a same species occur in different proportions depending on the sample, suggesting that each sample has its own subtypes community. Phylogenetic analysis revealed a large variety of *Malassezia* subtypes, indicating intra-specific diversity.

The calculated genetic diversity per locus (FST) values for the three populations of *M. globosa* strains ranged from 0.018 to 0.057, indicating weak differentiation between them. This result was supported by the MStree, showing that multilocus microsatellite genotypes contains isolates collected from breeders and patients, corresponding to human isolates were not separated from animal (dogs) ones. A dominant clonal mode of reproduction in *M. globosa* could be implicated. But also, genetic population structure analysis of human (patients and breeders) and animal *Malassezia* isolates carried out in a restricted geographic area could be suggested.

## 5. Conclusions

*Malassezia* genus have been widely studied in recent years due to their association with skin diseases. Different aspects have been analyzed, such as the specific fungal roles on the pathogenic process and the host immune response. The genomic sequences of *M. globosa* and *M. sympodialis* have been completely determined and the partial sequence of *M. restricta* is also available. Despite advances, little is still known concerning *Malassezia* genetic diversity. In this study, we describe the successful development of microsatellite PCR for genotyping *M. globosa* and *M. restricta* isolates from different sources and the subsequent application of this method to an extended number of other *Malassezia* species of clinical interest. Moreover, this method is a valuable tool for epidemiological studies, for the kinetics knowledge of the colonization-to-infection process and for the study of genotype’s antifungal susceptibility.

## Figures and Tables

**Table 1:** Features of the polymorphic VNTR of *M. globosa* and *M. restricta* upon analysis of 68 isolates.

Species	Marker Name	Primer Sequences (5′ to 3′)	DNA Motifs (bp)	Number of Alleles	Fragment Size (bp)	Simpson Diversity Index D	Marker Location
*M. globosa*	STR-MG1	**F: FAM**-CACGCTAAACTGCTGGATGA	2	6	242-256-270-286-296-302	0.829	Ch9
		R: CGAATATGAACCCCATGGAC					
	STR-MG2	**F: HEX**-CGGTAGTACGATTGGCCCTA	2	5	198-228-232-272-282	0.741	Ch3
		R: CCACATCATACGAGCCACAC					
	STR-MG3	**F: TET**-GGATGAAGAGGCTCGCTATG	3	5	168-177-189-213-234	0.722	Ch3
		R: ACAGGCGTAGAAGCCAAAGA					
	STR-MG4	F: GGCGGTGGTAAGCACTCTGG	66	6	250-300-350-400-450-500	0.807	Ch1
		R: ATGGGTGGTCACCACATGG					
	STR-MG5	F: AGGGGCTTCACGGTCATGTG	86	4	300-400-500-700	0.649	Ch3
		R: CATTGGAGCCTGGTGTGACG					
	STR-MG6	F: AACCAACGACGTCCAGAAAC	176	3	300-450-700	0.581	Ch5
		R: GTTAAGCTCGCTTGCTCGAAT					
*M. restricta*	STR-MR1	**F: FAM**-TAGCACTATCCCAACGTACC	2	5	242-250-254-258-286	0.774	Ch7
		R: GTTCCGTGTGTCATGATTGC					
	STR-MR2	**F: HEX**-CGCAGAAAACCTAGAGACG	3	5	258-264-288-297-336	0.818	Ch3
		R: CTCGTGCGTGTGAGTATTGG					
	STR-MR3	**F: TET**-GCCTTGGATGCACTGGTATT	3	4	278-314-338-341	0.642	Ch2
		R: CACGGCGTCAGGAACAAGAG					
	STR-MR4	F: ACGGGTTCGAACGGTGAG	66	5	250-350-450-500-600	0.775	Ch4
		R: ATGATTGCTTGCGTTGACTG					
	STR-MR5	F: TATGGGTGCTGCCAGAGTCG	162	3	300-600-750	0.549	Ch4
		R: GTCGAAGGAGATTCACGGCG					
	STR-MR6	F: CCCACCACCAACTAACAACA	228	3	250-500-750	0.521	Ch5
		R: AAACACGGACCACACAACAC					

**Table 2:** *M. globosa* F_st_ values comparing patients, breeders and animals genotypes.

	*Pop E*	*Pop A*
***Pop P***	0.018	0.045
***Pop E***		0.057

Pop P: population of patients; Pop E: population of breeders; Pop A: population of animals (Dogs).

**Table 3:** Association of covariates with the ten *Malassezia* genetic clusters identified by minimum spanning tree (MStree) analysis.

Cluster (%)	I	II	III	IV	V	VI	VII	VIII	IX	X	Singleton	*p*
Patient	-	15.38	7.69	-	3.84	3/26	7.69	7.69	11.53	7.69	26.92	>0.05
Breeder	11.11	11.11	-	-	11.11	33.33	-	-	-	11.11	11.11	0.16
Dog	22.22	33.33	-	11.11	11.11	11.11	-	-	-	-	11.11	0.16
Global Site effect												>0.05
*Malassezia* folliculitis		25	12.5	-	-	6.25	12.5	12.5	-	12.5	18.75	-
Pityriasis versicolor		-	-	-	22.22	22.22	-	-	33.33	11.11	11.11	-
Pityriasis capitis		-	-	-	20	20	-	-	-	-	60	0.01
Healthy human	25	-	-	25	-	25	-	-	-	-	25	-
Global Year effect												<0.05
2016	13.63	18.18	-	9.09	18.88	13.63	-	-	-	9.09	18.88	-
2017	-	-	-	-	-	40	-	40	-	-	20	-
2018	-	23.52	11.67	-	-	5.88	11.67	-	17.64	5.88	23.52	-

## Data Availability

All data are available within the manuscript.
